# Comorbid depressive disorders and left‐side dominant occlusal discomfort in patients with phantom bite syndrome

**DOI:** 10.1111/joor.12872

**Published:** 2019-08-28

**Authors:** Yukiko Shinohara, Yojiro Umezaki, Ichiro Minami, Motoko Watanabe, Anna Miura, Lou Mikutsuki, Kaoru Kawasaki, Shiori Sugawara, Tu Thi Hyen Trang, Takayuki Suga, Takeshi Watanabe, Tatsuya Yoshikawa, Miho Takenoshita, Haruhiko Motomura, Akira Toyofuku

**Affiliations:** ^1^ Department of Psychosomatic Dentistry, Graduate School of Medical and Dental Sciences Tokyo Medical and Dental University Tokyo Japan; ^2^ Section of Geriatric Dentistry, Department of General Dentistry Fukuoka Dental College Fukuoka Japan; ^3^ Department of Removable Partial Prosthodontics, Masticatory Function Rehabilitation, Graduate School Tokyo Medical and Dental University Tokyo Japan; ^4^ Department of Oral and Maxillofacial Radiology Tokyo Dental College Tokyo Japan

**Keywords:** central dysfunction, occlusal dysesthesia, phantom bite syndrome, psychiatric disorders, symptomatic area

## Abstract

**Background:**

Phantom bite syndrome (PBS) is characterised by occlusal discomfort without corresponding dental abnormalities. Despite repeated, failed dental treatments, patients with PBS persist in seeking bite correction. PBS has been regarded as a mental disorder. However, we have reported that PBS patients with a dental trigger tend to have less psychiatric history than those without. Hence, the symptoms of PBS cannot be explained by a mental disorder alone, and it is unclear if mental disorders affect occlusal sensation.

**Objective:**

To elucidate the pathophysiology of PBS, we analysed the dental history, PBS symptom laterality and psychiatric history of patients.

**Methods:**

In this retrospective study, we reviewed outpatients with PBS who presented at our clinic between April 2012 and March 2017. Their medical records were reviewed for demographic data, medical history and laterality of occlusal discomfort.

**Results:**

Approximately half of the 199 enrolled patients had bilateral occlusal discomfort. In the others, the side with occlusal discomfort generally tended to be the one that had received dental treatment. There was no significant relationship between the side chiefly affected by occlusal discomfort and whether dental treatment had been received; however, the affected side differed depending on whether the patient had comorbid psychiatric disorders (*P* = .041).

**Conclusions:**

The distributions of the side with symptoms of PBS were different between those with and without comorbid psychiatric disorders, suggesting that psychiatric disorders might affect occlusal sensation due to a subtle dysfunction in brain areas central to sensory integration. Central dysfunction might play an important role in PBS.

## 1 BACKGROUND

“Phantom bite syndrome” (PBS),^1^, ^2^ sometimes referred to as “occlusal dysesthesia”,^3^, ^4^ is characterised by an uncomfortable sensation mainly affecting a corrected dentition in which no abnormality can be clinically explained. Despite repeated failures of dental surgery, affected individuals persist in seeking bite correction from a succession of dentists.^5^ They become increasingly difficult to manage after repeated failures of dental surgery, resulting in frustration for both the dentist and patient, who is usually convinced of the incompetence of the dentist and moves on to another.

Since patients may complain of denture intolerance or have an obsessional concern about the inability to achieve comfortable occlusion, PBS is primarily regarded as a mental disorder in clinical settings. Marbach et al^6^ classified PBS as a monosymptomatic hypochondriacal psychosis and was supported by Clark et al^7^ Reeves et al^8^ identified PBS as a somatoform disorder using criteria in the fourth edition of the Diagnostic and Statistical Manual of Mental Disorders (DSM‐IV).^9^ Tsukiyama et al^10^ evaluated patients with occlusal dysesthesia using psychological tests and reported that these patients tended to have psychosomatic problems, such as somatic symptoms and depression. PBS is an uncommon manifestation; however, dentists are concerned mainly with the mechanical aspects of occlusion, adding further complications to PBS. In a previous study, we had reported that PBS patients with a dental trigger had significantly less psychiatric history than those without a dental trigger.^11^ Hence, the symptoms of PBS cannot be explained by a mental disorder alone. Furthermore, it is unclear if mental disorders affect occlusal sensation.

An analysis of the correspondence between the symptomatic area and the area of previous dental treatment for PBS would clarify whether the dento‐maxillofacial system innervated by the trigeminal nerves was limited. When the distribution of the former areas corresponds to that of the latter areas, the pathophysiology of PBS might be explained by the limited dento‐maxillofacial system, and vice versa. Comparison of the distributions of such areas between PBS patients with and without various mental disorders is one way to test how mental disorders affect occlusal sensation. Therefore, an analysis of correspondence as described above contributes to the elucidation of the pathophysiology of PBS in light of neural circuits. However, so far, there have been no reports on the relationship between the symptomatic area and the area that required dental treatment on the basis of comorbid mental disorders. Therefore, the purpose of this retrospective study was to investigate the role of dental invasion as a trigger for PBS, while analysing the patients’ dental history, laterality of the PBS symptoms and psychiatric history.

## 2 MATERIALS AND METHODS

### 2.1 Enrolment of patients with phantom bite syndrome

This retrospective study reviewed consecutive outpatients with PBS treated in the department of psychosomatic dentistry in Tokyo Medical and Dental University (TMDU) between April 2012 and March 2017. This study was conducted with the approval of the Ethical Committee of TMDU (No. 356). The department of psychosomatic dentistry in TMDU is a specialised clinic for oral psychosomatic problems such as burning mouth syndrome, atypical odontalgia, oral cenesthopathy and PBS. Almost all patients were referred from general physicians/dentists or another department in TMDU.^12^ Among 2877 first‐visit patients during the 5 years in our clinic, 226 were diagnosed with PBS, of which 83 patients had started treatment with us.

All participants in this study had been diagnosed with PBS by a specialist in psychosomatic dentistry, based on the criteria suggested by Melis,^3^ as follows: (a) complaints of an uncomfortable sensation while biting; (b) significantly associated emotional distress; (c) symptoms lasting more than 6 months; (d) history of failures of various bite‐altering dental procedures; (e) absence of dental occlusion discrepancies or those disproportional to the complaint; and (f) not attributed to another disorder (dental, pathology, muscle, temporomandibular joint or neurological disorder). Our data included patients who had been analysed in one of our former studies.^11^ Exclusion criteria were the presence of a history of organic brain disorder, obvious neurological disorders in the trigeminal nerves and patients with communication difficulties.

### 2.2 Data collection

We collected patient demographic data, medical history and details of symptoms from medical records and interviews at the first visit. The Zung self‐rating depression scale (SDS)^13^ was used for every patient to survey symptoms of depression. The subjective severity of uncomfortable bite sensation was assessed using a visual analogue scale (VAS). The laterality of the symptoms was classified into three groups: “right side”, “left side” and “both sides”. The psychiatric diagnoses were collected from the referral letters of the patients’ psychiatrists. Psychiatric disorders were categorised according to the Diagnostic and Statistical Manual of Mental Disorders, 5th Edition (DSM‐5).^14^

### 2.3 Statistical analysis

Data were analysed with the chi‐square test using PASW software (IBM). *P*‐values < .05 were considered to indicate statistical significance.

## 3 RESULTS

### 3.1 Demographic data and laterality of the symptoms

Among a total of 199 patients with PBS who enrolled in this study, 163 and 36 patients were female and male, respectively. The mean age was 53.48 years old. Male patients were significantly younger than female patients (48.47 and 54.59 years old, *P* = .027). According to the laterality of the occlusal symptoms, patients were classified into three groups: “both sides” (112 patients, 56.28%), “left side” (39 patients, 19.60%) and “right side” (48 patients, 24.12%). The distributions of sexes did not differ between these groups. In addition, the SDS score, VAS score and duration of illness were not significantly different between the three groups (Table 1).

**Table 1:** Demographic data of patients with phantom bite syndrome

	Side of the symptom			
	“Left side”	“Both sides”	“Right side”	Total
Number of patients, total (%)	39 (19.6%)	112 (56.28%)	48 (24.12%)	199
Female (%)	32 (19.63%)	89 (54.6%)	42 (25.77%)	163
Male (%)	7 (19.44%)	23 (63.89%)	6 (16.67%)	36
SDS score	46.1795	48.4909	46.3542	48.1035
VAS score	33.5135	37.8102	43.6875	44.3276
Duration of illness (mo)	75.5789	69.9722	70.5417	65.6724
Triggers				
Dental treatment	31	85	39	155
Left side	24	15	2	41
Left and right side	6	62	14	82
Right side	1	8	23	32
Other than dental treatment	8	27	9	44

Abbreviations: SDS, self‐rating depression scale; VAS, visual analogue scale.

### 3.2 Consistency between dental triggers and PBS symptom laterality

Among a total of 199 patients with PBS, 44 patients developed PBS without dental treatment (psychosocial stress, 19 patients; spontaneous, 16 patients; physical illness, 4 patients; other, 5 patients). In order to verify the possibility that the laterality of symptoms was affected by the dental treatment itself, we analysed the relationship between symptom laterality and the trigger (presence or absence of dental treatment). We observed that symptom laterality was not significantly related to dental treatment in either group (*P* = .726). However, the side of dental treatment corresponded with the symptomatic side of PBS in many cases (left‐sided treatment and “left side”, 24 cases; bilateral treatment and “both sides”, 62 cases; right‐sided treatment and “right side”, 23 cases). In some patients, “both sides” symptoms developed after unilateral dental treatment (left‐sided treatment and “both sides”, 15 cases; right‐sided treatment and “both sides”, eight cases), and “right side” or “left side” symptoms developed after bilateral dental treatment (bilateral treatment and “left side”, six cases; bilateral treatment and “right side”, 14 cases). Only a few patients developed “right side” or “left side” symptoms triggered by contralateral dental treatment (left‐sided treatment and “right side”, two cases, right‐sided treatment and “left side”, one case). Therefore, the side affected by PBS symptoms corresponded to or included the side that received dental treatment. “Both sides” were more frequent than “right side” and “left side” among the 44 cases whose symptoms developed without dental treatment.

### 3.3 Comorbidity of psychiatric disorders and PBS symptom laterality

When all patients with PBS were classified into two groups, with or without comorbid psychiatric disorders, the distributions of “right side”, “left side” and “both sides” were significantly different (*P* = .041; Table 2). Then, we scrutinised the relationship between the laterality of the PBS symptoms and the presence of psychiatric disorders. As to the details of psychiatric disorders, depressive disorders were present in 36 patients, anxiety disorders in 26 patients, sleep‐wake disorders in 13 patients, bipolar and related disorders in 11 patients, somatic symptoms and related disorders in 9 patients, schizophrenia spectrum and other psychotic disorders in 6 patients, trauma‐ and stressor‐related disorders in 4 patients, feeding and eating disorders in 3 patients, neurodevelopmental disorders in 2 patients, obsessive‐compulsive and related disorders in 1 patient, personality disorders in 1 patient and unknown disorders, including multiple diagnoses, in 4 patients.

**Table 2:** The number of patients of each symptom laterality divided according to comorbid psychiatric disorders

	Side of the symptom		
	“Left side”	“Both sides”	“Right side”
No history of psychiatric disorders	11	54	29
History of psychiatric disorders, total^a^, *	28	58	19
Depressive disorders*	15	19	2
Anxiety disorders	4	17	5
Sleep‐wake disorders	5	6	2
Bipolar and related disorders	4	5	2
Somatic symptom and related disorders	3	4	2
Schizophrenia spectrum and other psychotic disorders	0	4	2
Others^b^	2	5	7
Total	39	112	48

a Includes multiple diagnoses.

b Others include trauma‐ and stressor‐related disorders, feeding and eating disorders, neurodevelopmental disorders, obsessive‐compulsive and related disorders, personality disorders and unknown cases.

* The distributions of side of the symptom were significantly different from that of “no history of psychiatric disorders”. (*P* < .05).

Compared to the 94 patients without any history of psychiatric disorders, the 26 patients with histories of anxiety disorders showed a similar distribution of symptom laterality (*P* = .494). However, the 36 patients with histories of depressive disorders did not show a similar distribution (*P* < .001), more frequently reporting “left side” symptoms (“both sides”, 19 cases; “left side”, 15 cases; “right side”, 2 cases). This tendency may also be common in patients with bipolar disorders; however, there were an insufficient number of cases to analyse this fully. In contrast, in the 6 PBS patients with schizophrenia, 4 patients reported “both sides”, 2 patients “right side” and no patients reported “left side”.

The distribution of symptom laterality in patients with a depressive history was significantly different from the patients without any history of psychiatric disorders (*P* < .001). The details of symptom laterality and dental triggers in the 36 patients with depressive history are shown in Table 3. Similar to other patients in the current study, the symptomatic side tended to reflect the side of the dental treatment. However, in the patients who developed symptoms without a dental trigger, the symptom laterality was more frequently “left side” than “right side” (“left side”, 4 patients; “both sides”, 4 patients; and “right side”, no patients).

**Table 3:** Details of the symptomatic side and site of triggering dental treatment in 36 patients with depressive disorders

	Side of the symptom			
	“Left side”	“Both sides”	“Right side”	Total
Site of dental treatment				
Left	8	2	0	10
Left and right	3	11	1	15
Right	0	2	1	3
Nothing	4	4	0	8

## 4 DISCUSSION

In this retrospective study, patients with PBS were classified into three groups: symptoms on “both sides”, symptoms on the “left side” and symptoms on the “right side”, according to the laterality of the occlusal symptoms. Although many classifications for the symptomatic area exist, we classified them into three groups because of the limited number of participants. The six primary results are detailed below.

1. The “both sides” group included most of the patients with PBS.
2. The trigger of the PBS symptoms was dental treatment in around 75% of cases, and the side of dental treatment tended to be consistent with PBS symptom laterality.
3. PBS symptom laterality was not affected by the presence or absence of a dental trigger.
4. Furthermore, the distributions of the three groups were different between patients with and without a psychiatric history.
5. “Left side” symptoms were more common in the PBS patients with depression.
6. The shift in the distribution of the laterality in the PBS patients with depression was common regardless of the presence of a dental trigger.

### 4.1 Dominance of bilateral symptoms in the PBS patients

Among 199 patients with PBS, over half of the patients had bilateral symptoms. The patients with bilateral symptoms were more common in this study. This tendency was common regardless of whether PBS was triggered by dental treatment. The reason why bilateral symptoms were more common remains unclear; however, the fact that bite sensation on one side is dependent on the opposite side could affect this distribution. Considering that bilateral symptoms are more common, unilateral PBS symptoms seem to be notable and also indicate that affected patients had limited dysfunction in the occlusal system. The heterogeneity of PBS might emerge with respect to the symptomatic side, and more subdivided heterogeneity might also exist in the bilateral symptom patients.

### 4.2 Possible right hemisphere dysfunction in PBS patients

In this study, the side of the PBS symptoms corresponded to the side that received dental treatment; however, the ratio of “right side” symptoms, “left side” symptoms and “both sides” symptoms was not influenced by the nature of the triggers. These data might imply a hypothesis that the patients originally had some diathesis for developing PBS symptoms.

Neural mechanisms underlying PBS seem to be quite complicated. The results in this study suggest that deviation of PBS symptom laterality cannot simply be explained in terms of trigeminal nerve function. Not only the trigeminal sensory process, but also the association cortex responsible for higher cognitive function might be involved.

According to previous reports, experimentally reproduced occlusal discomfort seems to be related to the right frontal area, as suggested by a near‐infrared spectroscopy‐based study.^15^ On the other hand, the human right hemisphere plays a dominant role in corporeal awareness, possibly through a specific branch of the superior longitudinal fasciculus fibre tracts.^16^ Patients with PBS might have a dysfunction in corporeal awareness of the oral area, including the teeth and jaws. In this way, many brain areas above the trigeminal nerve may be involved in the pathophysiological mechanisms of PBS symptoms.

### 4.3 PBS symptom laterality and depressive history

The distribution of the symptomatic side in PBS patients with no psychiatric history was similar to that in patients with anxiety disorders, but different from that in patients with depressive disorders. The ratio of “both sides” symptoms was not different between patient groups; however, “left side” symptoms were more common in the patients with depressive disorders. This tendency remains regardless of whether the symptoms developed after dental treatment. The frequency of “left side” symptoms might be affected by central nerve system dysfunction associated with depressive disorders.

A lot of brain imaging studies for patients with depression have reported blood flow changes in various portions of the brain.^17^, ^18^ However, these results were not necessarily consistent, partly due to the heterogeneity in depression. Among them, asymmetry in grey matter volume reduction or cerebral blood flow between bilateral hemispheres has been reported.^19^, ^20^, ^21^

Our result showing the predominance of the “left side” over “right side” group in patients with depressive disorders might be due to a subtle dysfunction in brain areas central to sensory integration in the right hemisphere, resulting in the predominance of “left side” over “right side”.

Our study has some limitations. First, the psychiatric history was recorded only using the referral letter from the patients’ psychiatrists. Hence, some reporting biases may exist. However, when patients have a psychiatric history, the doctors in our clinic must contact the patients’ attending psychiatrists. Second, this study does not have a set control group. Since the main focus of this study was the relationship between the symptomatic side of PBS and other factors, we could not create a control group as would be possible in an interventional study focusing mainly on treatment effects. However, we believe that our findings will be useful as a reference for further studies to classify PBS into clinically valid subgroups.

## 5 CONCLUSION

We analysed patients with PBS, classifying them in accordance with their symptom laterality and psychiatric histories. A depressive history might be related to left‐sided occlusal discomfort in patients with PBS. In these cases, pre‐existing subtle central dysfunction might play a more important role than dental triggers. Additional studies and brain imaging analysis are warranted to investigate the features of the central nervous system in patients with PBS.

## CONFLICT OF INTERESTS

The authors declare that they have no competing interests.

## AUTHORS' CONTRIBUTIONS

YS, YU, HM and AT designed the study. YS, YU, MW, AM, LM, SS, KK, TT, TW, TS, TY and MT acquired the data. YS, YU, IM, HM and AT wrote the article. All authors analysed the data, reviewed the article and approved its publication.

## ETHICAL APPROVAL

This study was conducted with the approval of the Ethical Committee of Tokyo Medical and Dental University (No. 356).

## CONSENT FOR PUBLICATION

Consent for publication was obtained from every patient.

## Data Availability

The data sets used and/or analysed during the current study are available from the corresponding author on reasonable request.
